# Serum levels of interleukin-34 and RANKL as multivariable predictors of bone erosion seen by ultrasonography in patients with ankylosing spondylitis

**DOI:** 10.2478/abm-2022-0011

**Published:** 2022-04-29

**Authors:** Xianqian Huang, Yong Chen, Yong Peng, Minzhi Gan, Baoqing Geng, Mengya Zhu, Ying Ying

**Affiliations:** Department of Rheumatology, Ningbo Hwa Mei (No. 2) Hospital, Ningbo Institute of Life and Health Industry, University of Chinese Academy of Sciences, Ningbo, Zhejiang 315010, China

**Keywords:** enthesitis, interleukin-34 protein, human, receptor activator of nuclear factor-kappa B, spondylitis, ankylosing, ultrasonography

## Abstract

**Background:**

Ankylosing spondylitis (AS) is a chronic inflammatory arthritic disease, and sacroiliitis, enthesitis, and propensity for sacroiliac and spinal fusion are characteristic pathological features. Interleukin-34 (IL-34) plays a role in the induction and differentiation of osteoclasts. Other inflammatory factors are not directly involved in the induction and differentiation, but play an indirect role by modulating the level of receptor activator of nuclear factor-κB (RANKL) and other molecules during the process of inflammatory bone destruction in AS. However, to our knowledge, the relationship between enthesitis and bone erosion, and IL-34 and RANKL in AS has not yet been elucidated.

**Objective:**

To determine the correlation between serum IL-34, RANKL, and disease severity including enthesitis and bone erosion in patients with AS and develop multivariable predictive model.

**Methods:**

We conducted a cross-sectional study of 40 patients with AS, compared with 40 patients with osteoarthritis, and 40 healthy volunteers. Their serum levels of IL-34 and RANKL were measured using enzyme-linked immunosorbent assays (ELISAs). Enthesitis and bone erosion were assessed with real-time ultrasonography. Spearman rank correlation coefficients were determined to analyze the relationship between the variables. Multiple logistic regression was used to determine associations and receiver operating characteristic (ROC) curve analyses were conducted to determine the diagnostic performance of cytokine levels.

**Results:**

In patients with AS, serum levels of IL-34 (878.9 ± 116.4 pg/mL) and RANKL (155.6 ± 13.8 pg/mL) were significantly (*P* < 0.01) higher than those in patients with osteoarthritis (626.6 ± 79.0 and 138.1 ± 15.3 pg/mL, respectively) or a healthy group (612.9 ± 61.1 and 104.9 ± 15.4 pg/mL, respectively). Serum levels of IL-34 were not significantly correlated with the levels of RANKL. In patients with AS, serum levels of IL-34 and RANKL adjusted for age and weight were significantly correlated with enthesitis (0.798, *P* < 0.01; 0.347, *P* < 0.05, respectively) and bone erosion (0.822, *P* < 0.01; 0.368, *P* < 0.05, respectively). The area under the ROC curve (AUC) for the serum levels of IL-34 was 0.995 between patients with AS and healthy individuals. When serum level of IL-34 was >697.1 pg/mL, the sensitivity (SE) was >99% and specificity (SP) was 95.0%. The AUC for IL-34 was 0.982 between patients with AS and patients with osteoarthritis. When serum IL-34 was >688.4 pg/mL, the SE was >99% and SP 85.0%. IL-34 correlation with the number of bone erosions of enthesis was *r_s_* = 0.795, *P* < 0.01. The AUC for serum RANKL was 0.993 between patients with AS and healthy individuals. When serum RANKL was >126.2 pg/mL, the SE was 97.5% and SP 97.5%. The AUC for serum RANKL was 0.798 between patients with AS and patients with osteoarthritis. When serum RANKL was >149.3 pg/mL, the SE was 70% and SP was 80.0%.

**Conclusions:**

In patients with AS, serum levels of IL-34 and RANKL may be useful indicators of enthesitis, especially for bone erosions. IL-34 is associated with AS-associated enthesis damage and is a potential biomarker for predicting subsequent progression in patients with AS.

Ankylosing spondylitis (AS) is a chronic inflammatory arthritic disease, and sacroiliitis, enthesitis, and a marked propensity for sacroiliac joint and spinal fusion are its characteristic pathological features [1]. Inflammation, bone destruction, and new bone formation are the typical pathological changes during the pathogenesis of AS [2]. Lin et al. [3] identified inter-leukin-34 (IL-34) in 2008. Macrophage colony-stimulating factor (M-CSF) receptor is the target receptor for IL-34 [4], which plays a key regulatory role in the proliferation, differentiation, and survival of the mononuclear phagocyte lineage cells [4, 5]. In patients with rheumatoid arthritis, the synovium expresses IL-34, and high levels of IL-34 in synovial fluid (SF) and serum are associated with synovitis disease progression and severity [6,7,8]. The level of IL-34 in the SF and serum in patients with rheumatoid arthritis, and after treatment, the levels of IL-34 in SF and serum decreased [9]. SF levels of IL-34 correlated significantly with the symptomatic severity and radiographic of osteoarthritis of the knee [10]. IL-34 plays a role in the induction and differentiation of osteoclasts and it is a proinflammatory factor leading to bone destruction in patients with rheumatoid arthritis [11]. Other inflammatory factors are not directly involved in the induction and differentiation of osteoclasts, but play an indirect role by modulating the level of receptor activator of nuclear factor-κB (RANKL) and other molecules, such as tumor necrosis factor (TNF)-α and osteoprotegerin (OPG), during the process of inflammatory bone destruction in AS [12, 13].

Musculoskeletal ultrasonography is widely used to assess lesions in the tendons and joints of patients with AS. Because entheses are superficial and high-frequency grayscale ultrasonography has the advantage of high resolution, the operator can observe the slight changes in tendons, cartilage, and the bone surface of entheses. Compared with magnetic resonance imaging (MRI), musculoskeletal ultrasonography is sensitive to tendon structure, bursitis, and bone erosion and can easily detect the early calcification of entheses [14, 15]. Li et al. [16] used ultrasonography to show that the plasma IL-34 in patients with rheumatoid arthritis was correlated with RANKL and bone erosion and disease activity score. However, to our knowledge, the relationship between enthesitis and bone erosion, and IL-34 and RANKL in AS has not yet been elucidated. Therefore, we assessed enthesitis and bone erosion with ultrasonography and measured serum levels of IL-34 and RANKL using enzyme-linked immunosorbent assays (ELISA) to determine the correlations between serum levels of IL-34, RANKL, and enthesis lesions in patients with AS.

## Methods

After approval by the Ethics Committee of Ningbo Hwa Mei (No. 2) Hospital (reference No. PJ-NBEY-KY-2017-022-01), we conducted the present cross-sectional observational study in accordance with the principles of the contemporary amendment (2013) of the World Medical Association Declaration of Helsinki (1964), China Food and Drug Administration Good Clinical Practice for Drugs (GCP), Chinese Ministry of Health Measures for Ethical Review of Biomedical Research Involving Humans, and International Council of Medical Science Organizations International Biomedical Research Involving Humans. All patients and healthy volunteers provided freely-given written informed consent documented in writing. This article was prepared according to the transparent reporting of a multivariable prediction model for individual prognosis or diagnosis (TRIPOD) statement [17]. Statisticians estimated that we would require 40 participants in each arm based on a 95% 2-sided significance level and 80% power, assuming that 85% of participants finishing the study. The formula used for sample size calculation is published elsewhere [18].

We recruited 40 patients diagnosed with AS between August 2017 and November 2018, in accordance with the American College of Rheumatology (ACR) 1984 modification of the New York criteria [19]. Their Bath Ankylosing Spondylitis Disease Activity Index (BASDAI) was 4. AS patients had not yet accepted any intra-articular injection of any glucocorticoid, and they had no infection. We excluded tumor, osteoporosis, and other autoimmune diseases such as rheumatoid arthritis. We compared the serum levels of cytokines between these patients with AS, 40 patients with osteoarthritis, and 40 healthy volunteers. The diagnosis of osteoarthritis was made according to the classification of osteoarthritis by the American College of Rheumatology and the European League Against Rheumatism (EULAR). The patients included those with osteoarthritis of the knee and hand [20,21,22,23,24,25]. The patients with AS or osteoarthritis were from the outpatient and inpatient departments, and the healthy volunteers were from the physical examination center of Ningbo Hwa Mei (No. 2) Hospital, Zhejiang, PRC.

The clinical data included BASDAI, Bath Ankylosing Spondylitis Functional Index (BASFI), Bath Ankylosing Spondylitis Metrology Index (BASMI), Maastricht Ankylosing Spondylitis Enthesitis Score (MASES), Ankylosing Spondylitis Disease Activity Score (ASDAS), erythrocyte sedimentation rate (ESR), night visual analog scale (VAS) score, and C-reactive protein (CRP). Serum samples were prepared and stored at −80 °C until further use. No participants presented infectious disease, tuberculosis, hematologic disease, tumor, or osteoporosis. All healthy controls were confirmed as being without radiographic osteoarthritic changes on X-ray imaging before they were accepted as controls.

### Enzyme-linked immunosorbent assays

Human IL-34 and RANKL ELISA kits were from QiaoDu Biotechnology Co. (Shanghai, China). Serum levels of IL-34 and RANKL were measured by ELISA using a specific double-antibody sandwich method, according to the manufacturer's instructions. The serum samples for IL-34 and RANKL measurements were diluted 1:5. The intra- and interassay coefficients of variation (CV) were <15%, and the dilution recovery and recovery of authentic standards spiked in serum were 95.2 ± 2.7% and 90.6 ± 12.2%, respectively.

### Ultrasonography assessment

A skilled rheumatologist trained in musculoskeletal ultrasonography performed real-time ultrasonography using a Mylab One system (Esaote, Genova, Italy). Different investigators blinded to their mutual results performed the clinical examinations and the ultrasonography measurements. Sonographers were blinded to the patients’ clinical details. The monitoring positions referred to the common clinical enthesitis position and Glasgow Ultrasound Enthesitis Scoring System (GUESS) [26], including the patella–quadriceps tendon enthesis superior pole, the patella–proximal patellar ligament enthesis inferior pole, tibial tuberosity–distal patellar ligament enthesis, enthesis of the lateral femoral condyle, medial femoral condyle, medial tibial condyle, the superior pole of the calcaneus–Achilles tendon enthesis, and inferior pole of the calcaneus–plantar aponeurosis enthesis. In each participant, 18 entheses were evaluated. Inspection of the patellar ligament insertion at the tibial tuberosity, the inferior pole of the patella (patellar ligament origin), and the superior pole of the patella (quadriceps tendon insertion) was performed with the patient supine with their knee flexed at 30°. The Achilles tendon and the plantar aponeurosis were inspected with the patient prone and their feet placarding over the examination table edge at 90° of flexion. When the patient could not lie prone, their heels were inspected while they were supine and their ankles and knees were flexed at 90°. Ultrasonographic evaluation of bursitis, enthesophyte, bone erosion, and structure thickness was recorded at each site. The number of effusions, bone erosions, osteophytes of the enthesis, enthesitis, enthesis with power Doppler, and the GUESS score were recorded. To calculate the ultrasonography score of the lower limb enthesitis, for each site inspected, 1 point was scored for every abnormality, giving a maximum score of 36.

### Statistical analyses

All eligible patients during the study period were approached for participation. Participants with missing data were excluded. Statistical analyses were performed using IBM SPSS Statistics for Windows (version 17.0). MedCalc Software (Ostend, Belgium) was used to analyze the receiver operating characteristic (ROC) curves. Values are presented as means ± standard deviation (SD). A one-way analysis of variance (ANOVA) with least-squares difference (LSD) post hoc multiple comparison tests was used to examine continuous variables. A Fisher exact test or χ^2^ test was used to examine categorical variables. Spearman rank correlation coefficients were determined to analyze the relationship between the variables. Multiple logistic regression was performed to determine the associations between IL-34, RANKL, and disease parameters in patients with AS. Backward stepwise selection was applied to determine which variables were included in the final multivariable model. Several candidate predictors were selected to evaluate in the risk model, including IL-34, RANKL, weight, height, age, and sex. ROC curves were used to determine the diagnostic performance of serum levels of IL-34 and RANKL in patients with AS. *P* < 0.05 was considered significant.

## Results

### Characteristics of the participants

Ultimately, 120 participants were included in the present study. Their characteristics are shown in **Table 1**. Age, sex, height, and weight were matched between the AS, osteoarthritis, and healthy control groups. The past treatments of AS included nonsteroidal anti-inflammatory drugs (NSAIDs) (30%), TNF-α inhibitors (5%), sulfasalazine (15%), and methotrexate (2.5%). They had no current treatment at baseline.

**Table 1:** Characteristics of the participants

	**AS (n = 40)** **[A]**	**OA (n = 40)** **[B]**	**Healthy controls (n = 40)** **[C]**	***P*** **[A–B]**	***P*** **[A–C]**
Age (years)	44.5 ± 18.8	48.5 ± 8.6	46.2 ± 17.9	0.23	0.69
Height (cm)	164.7 ± 8.3	163.3 ± 7.4	165.7 ± 7.0	0.43	0.58
Weight (kg)	62.5 ± 6.5	62.5 ± 5.7	63.5 ± 7.0	0.96	0.53
Male (%)	40	20	35	0.051	0.64
Disease duration (years)	6.6 ± 6.7				
HLA-B27 (%)	100				
ESR (<20 mm/h)	49.3 ± 27.2				
CRP (<8.0 mg/L)	22.7 ± 16.8				
BASDAI	4.70 ± 0.69				
BASFI	4.75 ± 1.35				
MASES	2.70 ± 1.53				
ASDAS	3.27 ± 0.84				
Night VAS score	5.13 ± 1.74				
Clinical presence of enthesitis (%)	80				
(MASES ≥1) Dactylitis (%)	5				
Peripheral arthritis (%)	65				

One-way ANOVA with LSD post hoc multiple comparisons was used to examine continuous variables. A Fisher exact test or χ^2^ test was used to examine categorical variables.

ANOVA, analysis of variance; LSD, least-squares difference; AS, ankylosing spondylitis; OA, osteoarthritis; CRP, C-reactive protein; ESR, erythrocyte sedimentation rate; BASDAI, Bath Ankylosing Spondylitis Disease Activity Index; BASFI, Bath Ankylosing Spondylitis Functional Index; HLA-B27, human leukocyte antigen-B27; MASES, Maastricht Ankylosing Spondylitis Enthesitis Score; ASDAS, Ankylosing Spondylitis Disease Activity Score; VAS, visual analog scale.

### Serum levels of IL-34 and RANKL in patients with AS

**Figure 1:**
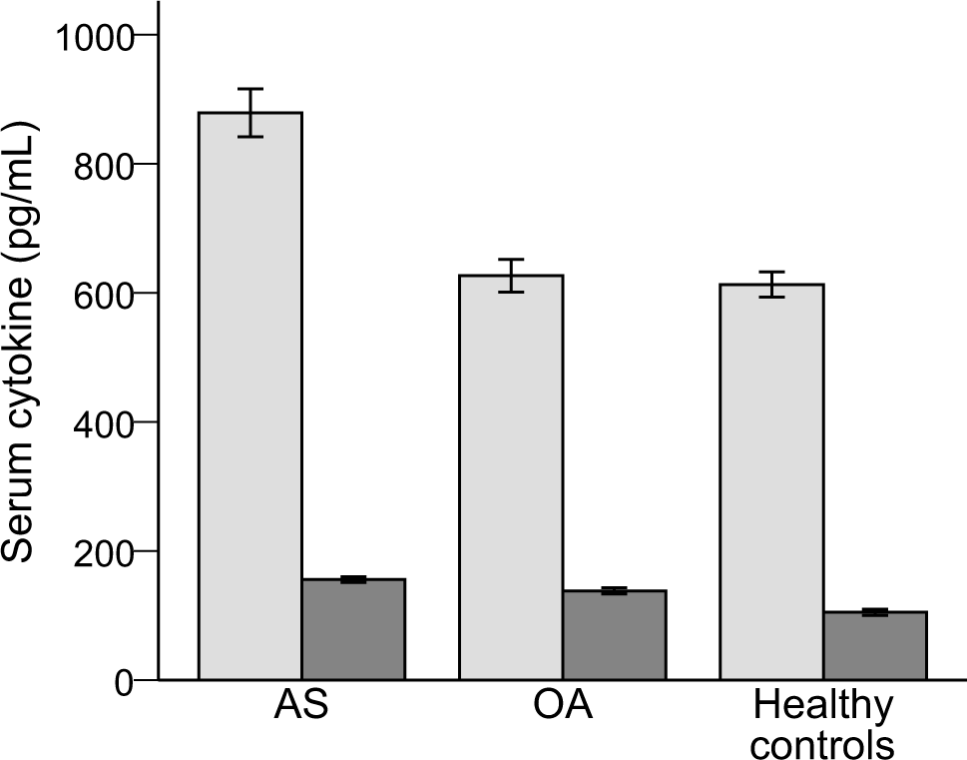
Serum levels of IL-34 and RANKL cytokines in AS, osteoarthritis, and healthy controls. IL-34, interleukin-34 (light-gray bars); RANKL, receptor activator of nuclear factor-κB (dark-gray bars); AS, ankylosing spondylitis; OA, osteoarthritis. Error bars indicate SD; SD, standard deviation.

Patients with AS had significantly higher serum levels of IL-34 (878.9 ± 116.4, *P* < 0.01) and RANKL (155.6 ± 13.8 pg/mL, *P* < 0.01) than patients with osteoarthritis (626.6 ± 79.0 and 138.1 ± 15.3 pg/mL respectively) or healthy controls (612.9 ± 61.1 and 104.9 ± 15.4 pg/mL, respectively) (**Figure 1**). Multiple logistic regressions were used to check the multicollinearity between serum levels of IL-34 and RANKL; however, we did not observe any multicollinearity in the model (condition index = 6.05, proportion of variation of IL-34 and RANKL was 0.05 and 0.08, respectively). Multivariable analyses showed that IL-34 [odds ratio (OR) = 0.93, 95% confidence interval (CI), 0.88–0.99, *P* = 0.016] was significantly associated with AS compared with OA. IL-34 (OR = 0.92, 95% CI, 0.87–0.98], *P* = 0.007) and RANKL (OR = 0.75, 95% CI, 0.64–0.89, *P* = 0.001) were both significantly associated with AS when compared with healthy controls (**Table 2**). **Tables 3** and **4** show that serum levels of IL-34 and RANKL did not vary significantly with age or body weight.

**Table 2:** Serum levels of IL-34 and RANKL in AS, osteoarthritis, and healthy control groups

**Comparison**	**Variable**	**Univariable analysis**			**Multivariable analysis**		
	
		**OR**	**95% CI**	***P***	**OR**	**95% CI**	***P***
AS vs. OA	IL-34	1.04	1.01–1.14	0.016	0.93	0.88–0.99	0.016
	RANKL	1.12	0.98–1.30	0.11	0.89	0.77–1.02	0.10
	Weight	0.43	0.12–1.58	0.20	0.67	0.63–8.57	0.20
	Height	1.17	0.57–2.37	0.68	0.86	0.42–1.74	0.67
	Age	0.88	0.88–1.00	0.06	1.14	0.999–1.30	0.052
	Sex	3.39	0.57–5.29	0.36	0.54	0.09–2.18	0.37

AS vs. Healthy controls	IL-34	1.24	0.80–1.32	0.11	0.92	0.87–0.98	0.007
	RANKL	0.82	0.41–1.18	0.45	0.75	0.64–0.89	0.001
	Weight	3.02	0.77–5.62	0.71	1.95	0.51–7.49	0.33
	Height	0.69	0.23–1.83	0.55	0.94	0.44–2.04	0.88
	Age	0.97	0.59–1.21	0.11	1.17	1.01–1.34	0.03
	Sex	0.40	0.10–2.18	0.36	0.51	0.03–2.31	0.60

AS, ankylosing spondylitis; OA, osteoarthritis; IL-34, interleukin-34; RANKL, receptor activator of nuclear factor-κB; CI, confidence interval; OA, osteoarthritis; OR, odds ratio.

Multiple logistic regressions were performed to determine the associations between IL-34, RANKL, and disease parameters in patients with AS. Backward stepwise selection was applied to determine which variables were included in the final multivariable model. Various candidate predictors were selected for evaluation in the risk model, including IL-34, RANKL, weight, height, age, and sex.

**Table 3:** Serum IL-34 and RANKL in patients with AS across age groups

**Age (years)**	**IL-34 (pg/mL)**	**RANKL (pg/mL)**
10 to <20	874.9 ± 164.5	154.0 ± 15.1
20 to <30	977.3 ± 91.2	136.4 ± 1.6
30 to <40	841.6 ± 126.1	157.6 ± 17.2
40 to <50	887.7 ± 105.2	159.7 ± 7.3
50 to <60	861.5 ± 97.0	145.7 ± 1.4
60 to <70	893.1 ± 116.4	162.0 ± 10.7
70 to 80	829.6 ± 60.5	155.8 ± 14.8

Mean ± standard deviation of serum levels of IL-34 and RANKL across age groups.

AS, ankylosing spondylitis; IL-34, interleukin-34; RANKL, receptor activator of nuclear factor-κB.

**Table 4:** Serum IL-34 and RANKL in patients with AS across weight categories

**Weight (kg)**	**IL-34 (pg/mL)**	**RANKL (pg/mL)**
40 to >50	984.5 ± 76.9	156.3 ± 13.6
50 to >60	881.3 ± 123.0	154.2 ± 15.5
60 to >70	846.5 ± 101.3	154.7 ± 12.7
70 to 80	990.0 ± 116.7	168.6 ± 4.2

Mean ± standard deviation of serum IL-34 and RANKL across weight categories.

AS, ankylosing spondylitis; IL-34, interleukin-34; RANKL, receptor activator of nuclear factor-κB.

### Ultrasonography

We examined 720 entheses in the 40 patients with AS. In 275/720 (38.2%) entheses, abnormalities were detected by ultrasound. The median GUESS score was 4 (range, 2–10). There were 82 effusions of entheses, 261 erosions of entheses, 111 osteophytes in entheses, and 73 entheses with power Doppler (PD) signals.

### Correlation between serum levels of IL-34, RANKL, and ultrasonography findings

**Figure 2:**
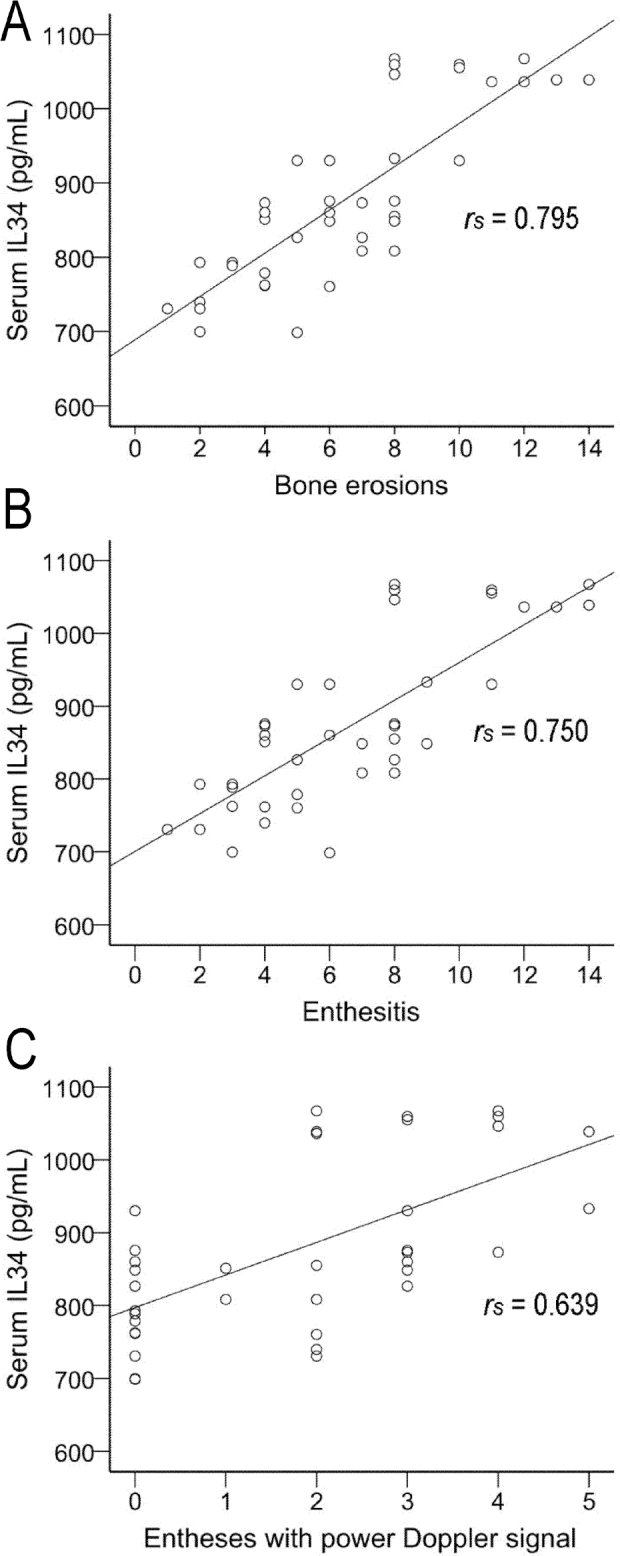
In patients with ankylosing spondylitis, the serum level of IL-34 was positively correlated with (**A**) the number of bone erosion, (**B**) the number of enthesitis, and (**C**) the number of entheses with a power Doppler signal. Spearman rank correlation coefficients between serum levels of IL-34 and clinical data were used to determine the relationship between the variables. IL-34, interleukin-34.

**Figure 3:**
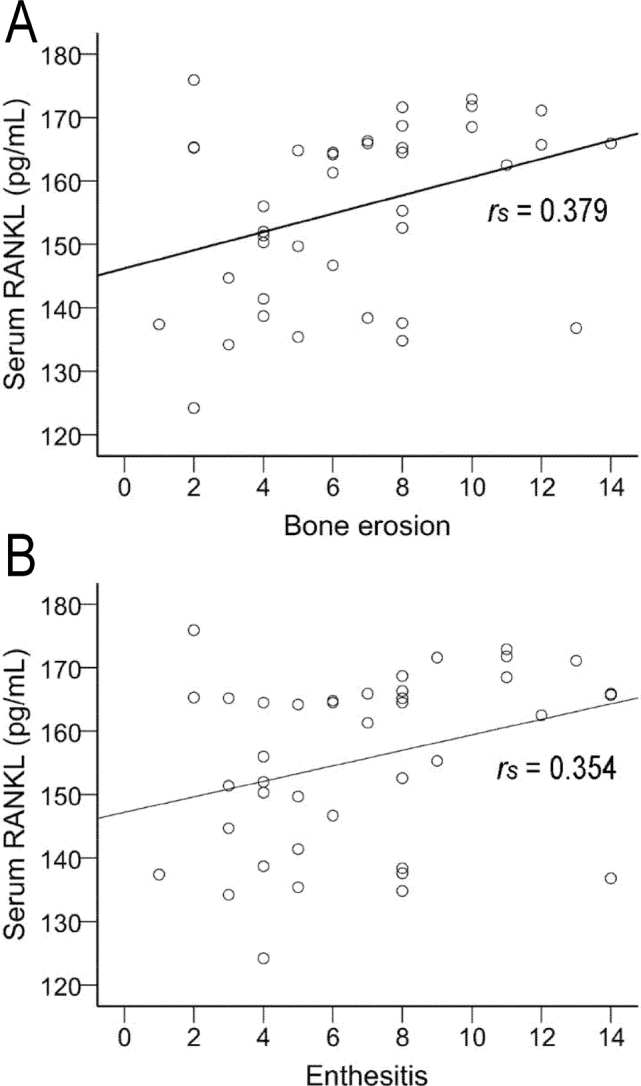
In patients with ankylosing spondylitis, the serum level of RANKL concentration was positively correlated with (**A**) the number of bone erosions and (**B**) the number of enthesitis. Spearman rank correlation coefficients between serum levels of IL-34 and clinical data were used to determine the relationship between the variables. RANKL, receptor activator of nuclear factor-κB.

In patients with AS, serum levels of both IL-34 and RANKL were correlated positively with enthesis bone erosion (**Figures 2A, B**) and enthesitis (**Figures 3A, B**), while only IL-34 was positively correlated with the number of entheses with a power Doppler signal (**Figure 2C**). In patients with AS, serum levels of IL-34 and RANKL after adjustment for age and weight also correlated positively with the numbers of enthesis bone erosion and entheses, while no correlation was observed for the effusions. Notably, after adjustment for age and weight, the serum levels of IL-34, but not RANKL, were positively correlated with the GUESS score, the number of osteophytes, and the number of entheses with PD (**Table 5**).

**Table 5:** Correlation (*r*_*s*_) between IL-34, RANKL, and ultrasonography findings in 40 patients with AS

**Ultrasonography**	**IL-34**	**RANKL**	**IL-34†**	**RANKL†**
GUESS	0.29	0.07	0.39^*^	0.26
Effusion	0.10	0.21	0.13	0.27
Bone erosion	0.80^**^	0.38^*^	0.82^**^	0.37^*^
Osteophyte	0.30	0.15	0.37^*^	0.13
PD	0.64^**^	0.07	0.63^**^	0.08
Enthesitis	0.75^**^	0.35^*^	0.80^**^	0.35^*^

* *P* < 0.05,

** *P* < 0.01.

† adjusted for age and weight.

Spearman rank correlation coefficients between serum levels of IL-34 or RANKL, and ultrasonography findings were used to determine the relationship between two variables.

AS, ankylosing spondylitis; PD, power Doppler signal; IL-34, interleukin-34; RANKL, receptor activator of nuclear factor-κB; GUESS, Glasgow Ultrasound Enthesitis Scoring System.

### Correlation between serum levels of IL-34 and RANKL and laboratory and clinical data

The correlation between the serum levels of IL-34 and RANKL was not significant. When adjusted for age and weight, correlations between IL-34, RANKL, ESR, CRP, BASDAI, BASFI, BASMI, MASES, ASDAS, and night VAS score were also not significant (**Table 6**).

**Table 6:** Correlation (*r*_*s*_) between serum IL-34 and RANKL, and clinical data of 40 patients with AS

**Clinical data**	**IL-34**	**RANKL**	**IL-34†**	**RANKL†**
RANKL	0.117	—	0.108	—
ESR	−0.056	0.206	−0.052	0.220
CRP	0.023	0.194	0.037	0.210
BASDAI	−0.211	0.213	−0.259	0.157
BASFI	−0.294	–0.089	−0.285	−0.149
BASMI	−0.254	0.27	−0.254	−0.149
MASES	0.078	−0.115	−0.012	−0.258
ASDAS	−0.274	0.281	−0.293	0.243
Night VAS	−0.282	0.202	−0.193	0.160

* *P* < 0.05,

** *P* < 0.01.

† adjusted for age and weight.

Spearman rank correlation coefficients between serum levels of IL-34 or RANKL, and clinical data were used to determine the relationship between two variables.

AS, ankylosing spondylitis; CRP, C-reactive protein; ESR, erythrocyte sedimentation rate; BASDAI, Bath Ankylosing Spondylitis Disease Activity Index; BASFI, Bath Ankylosing Spondylitis Functional Index; MASES, Maastricht Ankylosing Spondylitis Enthesitis Score; ASDAS, Ankylosing Spondylitis Disease Activity Score; VAS, visual analog scale; RANKL, receptor activator of nuclear factor-κB; BASMI, Bath Ankylosing Spondylitis Metrology Index; IL-34, interleukin-34.

### ROC curve analysis

**Figure 4:**
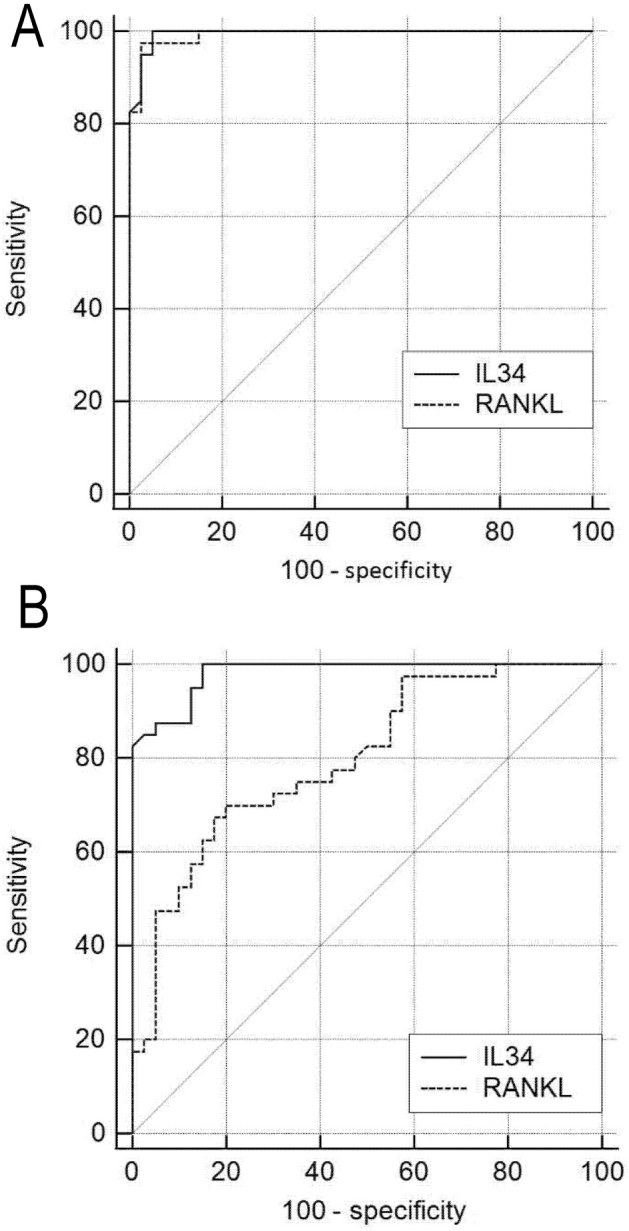
ROC curves for (**A**) serum level of IL-34 (solid line) and RANKL (dashed line) in the patients with AS and healthy individuals and (**B**) for serum level of IL-34 (solid line) and RANKL (dashed line) in patients with AS and those with osteoarthritis. ROC, receiver operating characteristics; IL-34, interleukin-34; RANKL, receptor activator of nuclear factor-κB; AS, ankylosing spondylitis; OA, osteoarthritis.

ROC curves were constructed for the AS, healthy control, and osteoarthritis groups. The areas under the ROC curves (AUCs) for the serum levels of IL-34 and RANKL were 0.995 (95% CI, 0.945–1.000, *P* < 0.01) and 0.993 (95% CI, 0.941 to >0.999, *P* < 0.01), respectively, when comparing the patients with AS with healthy individuals (**Figure 4A**). When the serum levels of IL-34 were >697.1 pg/mL, the sensitivity (SE) and specificity (SP) were maximal (SE 100%, SP 95.0%). When the serum levels of RANKL were >126.2 pg/mL, the SE and SP were maximal (SE 97.5%, SP 97.5%). The AUCs for the serum levels of IL-34 and RANKL were 0.982 (95% CI, 0.923–0.999, *P* < 0.01) and 0.798 (95% CI, 0.694–0.880, *P* < 0.01), respectively (**Figure 4B**). When the serum levels of IL-34 were >688.4 pg/mL, the SE and SP reached a maximum level (SE 100.0%, SP 85.0%). When the serum levels of RANKL were >149.3 pg/mL, the SE and SP were maximal (SE 70%, SP 80.0%).

## Discussion

In patients with AS, we found serum levels of IL-34 were significantly elevated compared with those in patients with osteoarthritis, and in healthy controls, which suggests that IL-34 is associated with AS. Interestingly, IL-34 was prominently and positively associated with the number of bone erosions of entheses, the number of enthesis with PD signal, and the number of entheses, which indicates that IL-34 is correlated with entheses.

To our knowledge, the correlation between IL-34 and AS had not yet been assessed. Our results suggest that as a macrophage colony-stimulating factor, IL-34 is secreted by osteoblasts in the pathogenesis of AS and released into circulation, where it can stimulate the viability of monocytes and colony formation of macrophages, and play a key role in osteoclastogenesis. Moreover, IL-34 has a proinflammatory effect. All these effects could accelerate the process of local bone destruction in AS.

ROC curves were constructed for the AS, healthy, and osteoarthritis groups. The AUC for the serum levels of IL-34 was 0.995 between patients with AS and healthy individuals. When serum IL-34 was >697.1 pg/mL, the SE and SP were optimal. The AUC for IL-34 was 0.982 between patients with AS and patients with osteoarthritis. When serum IL-34 was >688.4 pg/mL, the SE and SP were maximal. IL-34 was correlated with the number of bone erosion of enthesis *r_s_* = 0.795, *P* < 0.01. Thus, serum levels of IL-34 could be used as indicator of enthesitis in patients with AS, especially for those with bone erosion.

The RANKL/nuclear factor-κB (RANK)/OPG system participates in local bone erosion in patients with rheumatoid arthritis and animal models of this disease. Activated T cells and synovial fibroblasts in the synovium in AS produce RANKL, which is associated with local bone erosion [27]. Mori et al. [28] used in situ hybridization and detected the expression of RANKL, RANK, and OPG in the local joints of mice in a type II collagen-induced model of arthritis. In the present study, we used ELISA to detect immunoreactivity the protein directly and found that the serum levels of RANKL in AS patients were notably higher than in patients with osteoarthritis and healthy individuals. A positive correlation was established with the numbers of bone erosions and enthesitis. The AUC for serum RANKL was 0.993 between patients with AS and healthy individuals. When serum RANKL was >126.2 pg/mL, the SE and SP were optimal. The AUC of serum RANKL was 0.798 between patients with AS and patients with osteoarthritis. When serum RANKL was >149.3 pg/mL, the SE and SP were maximal. These findings indicate that the serum levels of RANKL could be a reliable indicator of bone erosion, especially enthesitis.

In the present study, serum levels of IL-34 and RANKL did not correlate significantly with ESR, CRP, BASDAI, BASFI, BASMI, MASES, ASDAS, or night VAS score; therefore, they do not reflect directly the activity of AS. Meanwhile, serum levels of IL-34 and RANKL were both positively associated with the number of bone erosions of enthesis. Both proliferation and growth of osteoclast progenitor cells can be induced by IL-34, and IL-34 plays a vital role in RANKL ligand-induced osteoclastogenesis [29, 30]. This mechanism might explain the results observed in this study, at least in part. Nevertheless, of note, the serum levels of IL-34 were not significantly correlated with the serum levels of RANKL. Thus, we speculate that IL-34 might be directly involved in the proliferation and differentiation of osteoclasts in AS, which might be independent of RANKL, and these 2 cytokines can play a proinflammatory role in enthesis; thereby, promoting the occurrence of bone erosion, especially the local bone erosion seen in patients with AS.

Enthesitis is the basic pathological change found in those with AS. Chronic enthesis inflammation will lead to the progressive destruction of joint bone, resulting in functional deterioration. Therefore, early diagnosis and prevention of bone erosions and enthesitis are critical for treatment of AS. Predicting the severity of bone erosion and enthesitis is valuable in guiding individualized treatment. The results of the present study suggest that IL-34, an osteoclastogenic cytokine, plays an important role in AS-associated enthesis damage and is a potential biomarker for predicting subsequent progression in patients with AS. Some patients with AS have poor responses to traditional drugs and TNF-α inhibitor therapy in clinical practice. Further understanding of the role of IL-34 might provide new therapeutic approaches.

The present study is limited in that it was a cross-sectional study that showed association rather than causality. The lack of follow-up limits the explanation of the role of IL-34 as an index of advancement of bone erosions. We were not able to show whether the rise of serum levels of IL-34 is earlier than bone erosion in AS patients. Therefore, prospective research in erosion-negative AS is needed to determine whether IL-34 can predict bone erosion. The small sample size precluded any reliable multivariable analysis. Thus, the findings of the present study need confirmation and duplication in longitudinal study and larger population samples.

## Conclusions

In patients with AS, the serum levels of IL-34 and RANKL may be useful indicators of enthesitis, especially bone erosions. Additional studies to determine the underlying specific mechanism of IL-34 in the bone destruction of AS might provide new therapeutic targets and approaches to the treatment of AS, especially for the AS patients who fail to respond to current drugs including TNF-α inhibitor therapy.
